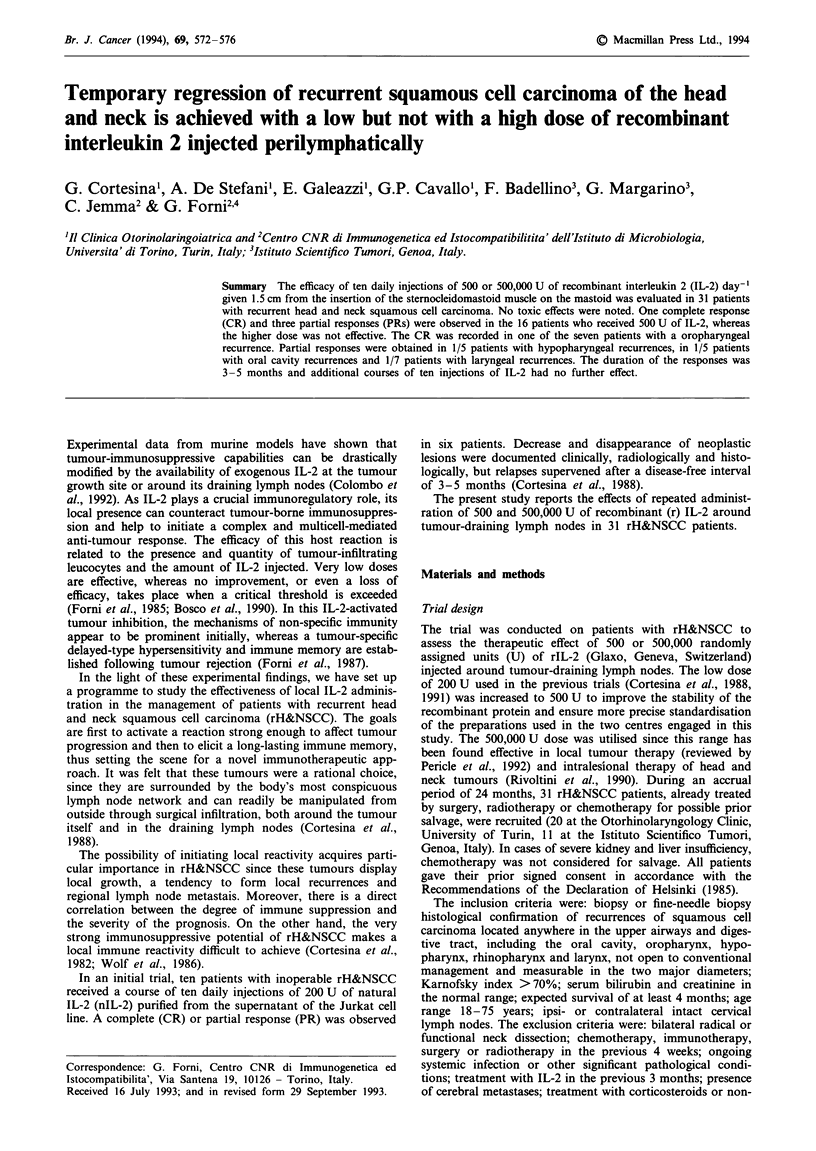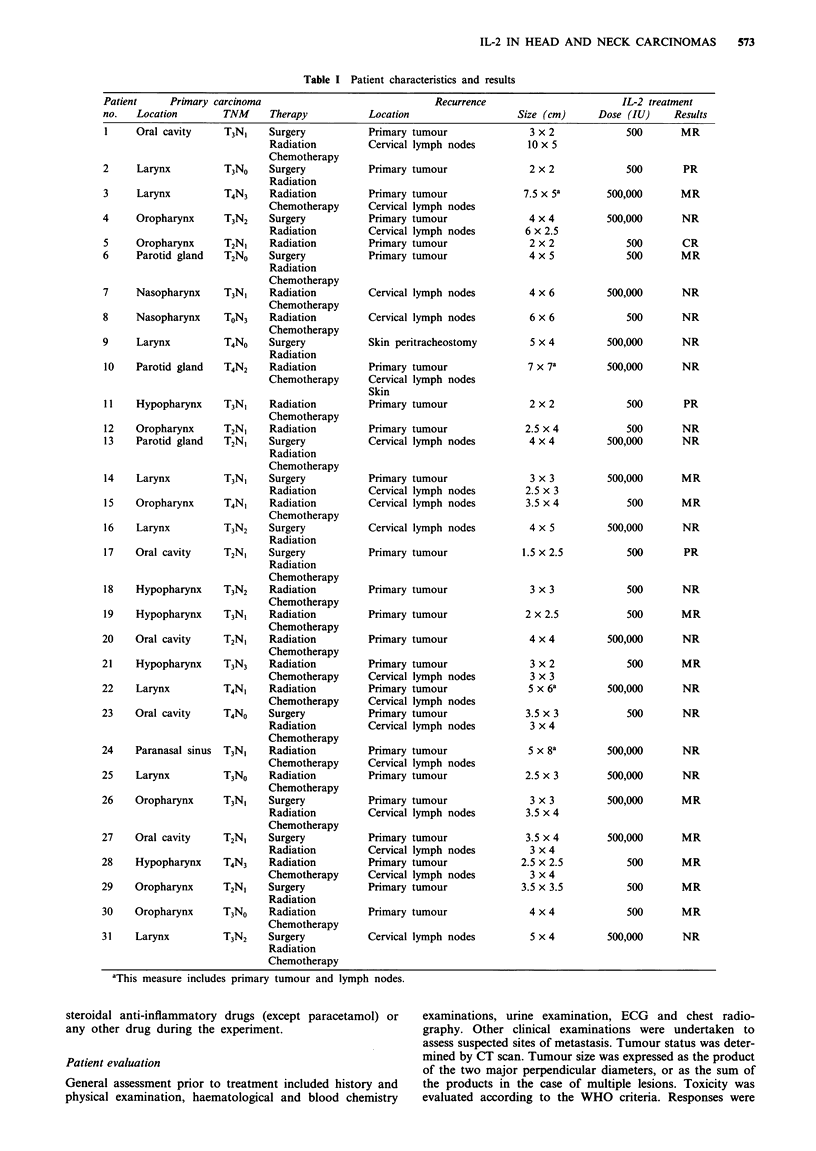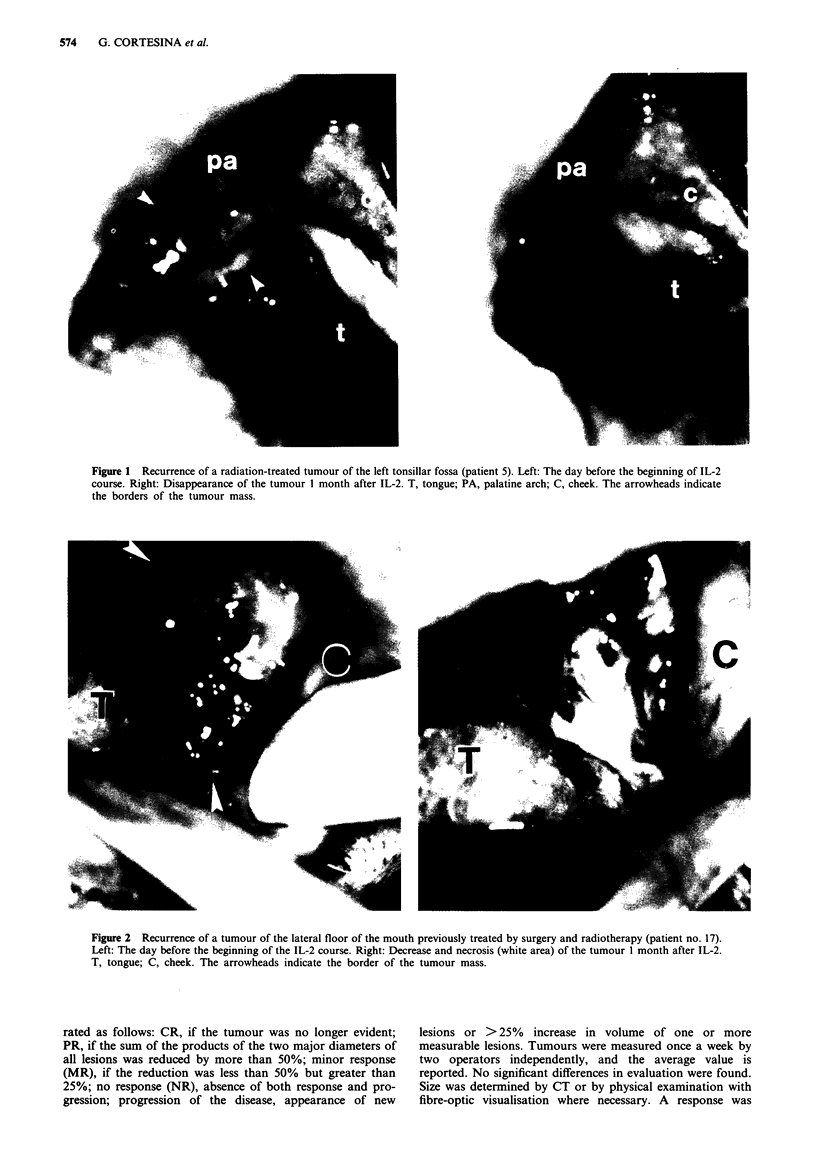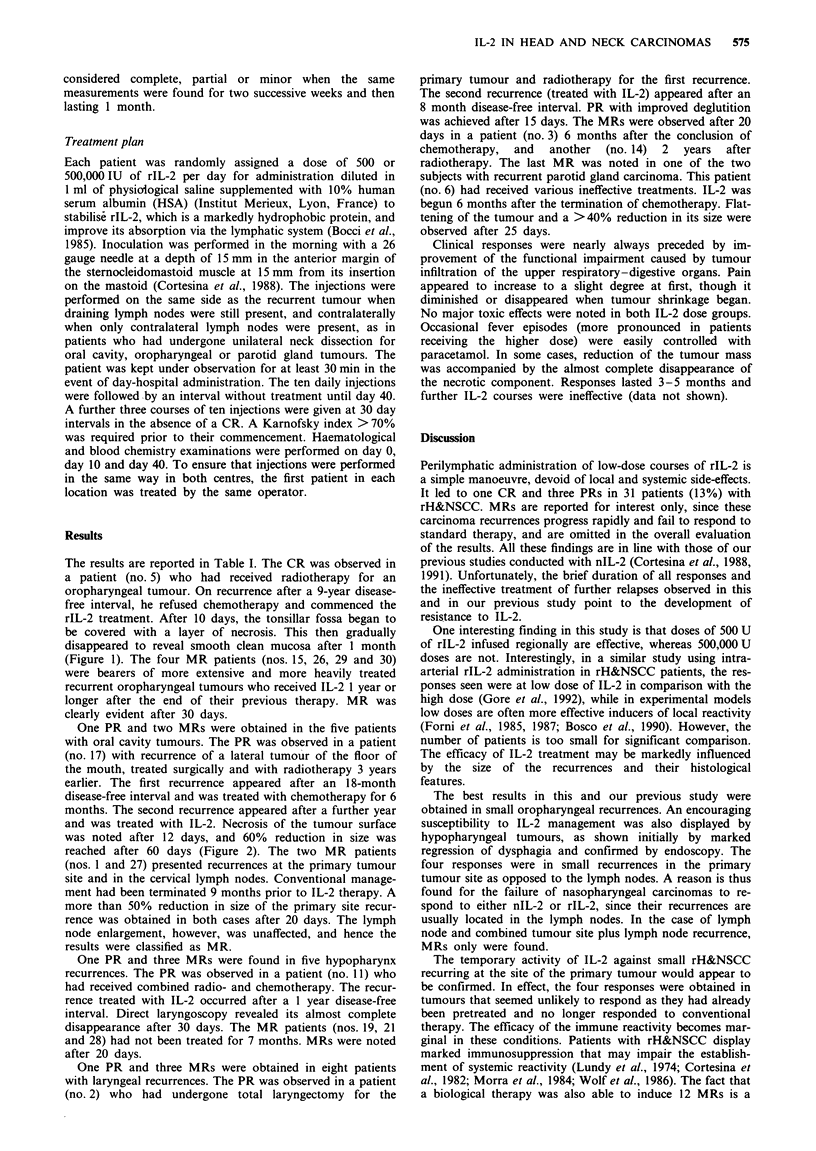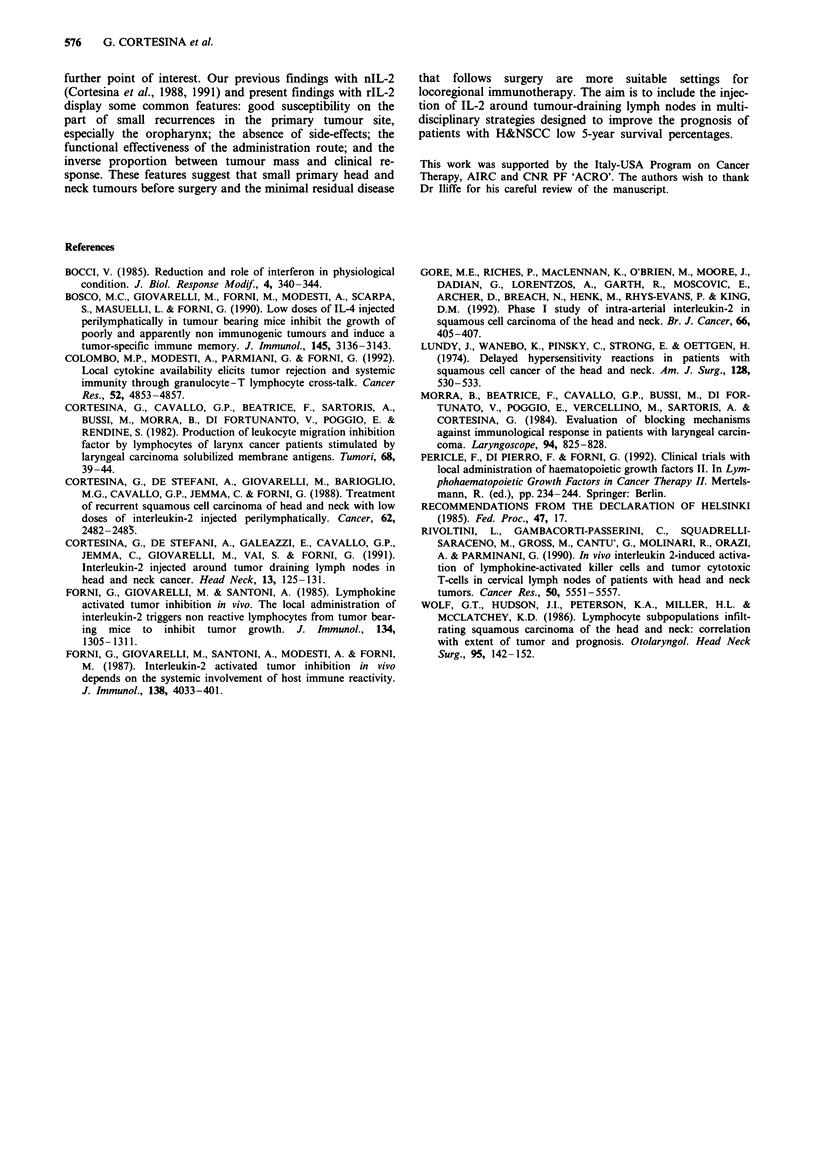# Temporary regression of recurrent squamous cell carcinoma of the head and neck is achieved with a low but not with a high dose of recombinant interleukin 2 injected perilymphatically.

**DOI:** 10.1038/bjc.1994.104

**Published:** 1994-03

**Authors:** G. Cortesina, A. De Stefani, E. Galeazzi, G. P. Cavallo, F. Badellino, G. Margarino, C. Jemma, G. Forni

**Affiliations:** II Clinica Otorinolaringoiatrica, Università di Torino, Turin, Italy.

## Abstract

**Images:**


Br. J. Cancer (1994), 69, 572–576 © Macmillan Press Ltd., 1994

### Temporary regression of recurrent squamous cell carcinoma of the head and neck is achieved with a low but not with a high dose of recombinant interleukin 2 injected perilymphatically

G. Cortesina[1], A. De Stefani[1], E. Galeazzi[1], G.P. Cavallo[1], F. Badellino[3], G. Margarino[3], C. Jemma[2] & G. Forni[2,4]

[1]*Il Clinica Otorinolaringoiatrica and* [2]*Centro CNR di Immunogenetica ed Istocompatibilitita' dell'Istituto di Microbiologia, Universita' di Torino, Turin, Italy;* [3]*Istituto Scientifico Tumori, Genoa, Italy.*

**Summary** The efficacy of ten daily injections of 500 or 500,000 U of recombinant interleukin 2 (IL-2) $day^{-1}$ given 1.5 cm from the insertion of the sternocleidomastoid muscle on the mastoid was evaluated in 31 patients with recurrent head and neck squamous cell carcinoma. No toxic effects were noted. One complete response (CR) and three partial responses (PRs) were observed in the 16 patients who received 500 U of IL-2, whereas the higher dose was not effective. The CR was recorded in one of the seven patients with a oropharyngeal recurrence. Partial responses were obtained in 1/5 patients with hypopharyngeal recurrences, in 1/5 patients with oral cavity recurrences and 1/7 patients with laryngeal recurrences. The duration of the responses was 3–5 months and additional courses of ten injections of IL-2 had no further effect.

Experimental data from murine models have shown that tumour-immunosuppressive capabilities can be drastically modified by the availability of exogenous IL-2 at the tumour growth site or around its draining lymph nodes (Colombo *et al.*, 1992). As IL-2 plays a crucial immunoregulatory role, its local presence can counteract tumour-borne immunosuppression and help to initiate a complex and multicell-mediated anti-tumour response. The efficacy of this host reaction is related to the presence and quantity of tumour-infiltrating leucocytes and the amount of IL-2 injected. Very low doses are effective, whereas no improvement, or even a loss of efficacy, takes place when a critical threshold is exceeded (Forni *et al.*, 1985; Bosco *et al.*, 1990). In this IL-2-activated tumour inhibition, the mechanisms of non-specific immunity appear to be prominent initially, whereas a tumour-specific delayed-type hypersensitivity and immune memory are established following tumour rejection (Forni *et al.*, 1987).

In the light of these experimental findings, we have set up a programme to study the effectiveness of local IL-2 administration in the management of patients with recurrent head and neck squamous cell carcinoma (rH&NSCC). The goals are first to activate a reaction strong enough to affect tumour progression and then to elicit a long-lasting immune memory, thus setting the scene for a novel immunotherapeutic approach. It was felt that these tumours were a rational choice, since they are surrounded by the body's most conspicuous lymph node network and can readily be manipulated from outside through surgical infiltration, both around the tumour itself and in the draining lymph nodes (Cortesina *et al.*, 1988).

The possibility of initiating local reactivity acquires particular importance in rH&NSCC since these tumours display local growth, a tendency to form local recurrences and regional lymph node metastais. Moreover, there is a direct correlation between the degree of immune suppression and the severity of the prognosis. On the other hand, the very strong immunosuppressive potential of rH&NSCC makes a local immune reactivity difficult to achieve (Cortesina *et al.*, 1982; Wolf *et al.*, 1986).

In an initial trial, ten patients with inoperable rH&NSCC received a course of ten daily injections of 200 U of natural IL-2 (nIL-2) purified from the supernatant of the Jurkat cell line. A complete (CR) or partial response (PR) was observed in six patients. Decrease and disappearance of neoplastic lesions were documented clinically, radiologically and histologically, but relapses supervened after a disease-free interval of 3–5 months (Cortesina *et al.*, 1988).

The present study reports the effects of repeated administration of 500 and 500,000 U of recombinant (r) IL-2 around tumour-draining lymph nodes in 31 rH&NSCC patients.

### Materials and methods

### Trial design

The trial was conducted on patients with rH&NSCC to assess the therapeutic effect of 500 or 500,000 randomly assigned units (U) of rIL-2 (Glaxo, Geneva, Switzerland) injected around tumour-draining lymph nodes. The low dose of 200 U used in the previous trials (Cortesina *et al.*, 1988, 1991) was increased to 500 U to improve the stability of the recombinant protein and ensure more precise standardisation of the preparations used in the two centres engaged in this study. The 500,000 U dose was utilised since this range has been found effective in local tumour therapy (reviewed by Pericle *et al.*, 1992) and intralesional therapy of head and neck tumours (Rivoltini *et al.*, 1990). During an accrual period of 24 months, 31 rH&NSCC patients, already treated by surgery, radiotherapy or chemotherapy for possible prior salvage, were recruited (20 at the Otorhinolaryngology Clinic, University of Turin, 11 at the Istituto Scientifico Tumori, Genoa, Italy). In cases of severe kidney and liver insufficiency, chemotherapy was not considered for salvage. All patients gave their prior signed consent in accordance with the Recommendations of the Declaration of Helsinki (1985).

The inclusion criteria were: biopsy or fine-needle biopsy histological confirmation of recurrences of squamous cell carcinoma located anywhere in the upper airways and digestive tract, including the oral cavity, oropharynx, hypopharynx, rhinopharynx and larynx, not open to conventional management and measurable in the two major diameters; Karnofsky index $>70\%$; serum bilirubin and creatinine in the normal range; expected survival of at least 4 months; age range 18–75 years; ipsi- or contralateral intact cervical lymph nodes. The exclusion criteria were: bilateral radical or functional neck dissection; chemotherapy, immunotherapy, surgery or radiotherapy in the previous 4 weeks; ongoing systemic infection or other significant pathological conditions; treatment with IL-2 in the previous 3 months; presence of cerebral metastases; treatment with corticosteroids or non-

Correspondence: G. Forni, Centro CNR di Immunogenetica ed Istocompatibilita', Via Santena 19, 10126 – Torino, Italy.
Received 16 July 1993; and in revised form 29 September 1993.

**Table I** Patient characteristics and results

| *Patient no.* | *Primary carcinoma Location* | *TNM* | *Therapy* | *Recurrence Location* | *Size (cm)* | *IL-2 treatment Dose (IU)* | *Results* |
|---|---|---|---|---|---|---|---|
| 1 | Oral cavity | $T_3N_1$ | Surgery<br>Radiation<br>Chemotherapy | Primary tumour<br>Cervical lymph nodes | 3 × 2<br>10 × 5 | 500 | MR |
| 2 | Larynx | $T_3N_0$ | Surgery<br>Radiation | Primary tumour | 2 × 2 | 500 | PR |
| 3 | Larynx | $T_4N_3$ | Radiation<br>Chemotherapy | Primary tumour<br>Cervical lymph nodes | 7.5 × 5[a] | 500,000 | MR |
| 4 | Oropharynx | $T_3N_2$ | Surgery<br>Radiation | Primary tumour<br>Cervical lymph nodes | 4 × 4<br>6 × 2.5 | 500,000 | NR |
| 5 | Oropharynx | $T_2N_1$ | Radiation | Primary tumour | 2 × 2 | 500 | CR |
| 6 | Parotid gland | $T_2N_0$ | Surgery<br>Radiation<br>Chemotherapy | Primary tumour | 4 × 5 | 500 | MR |
| 7 | Nasopharynx | $T_3N_1$ | Radiation<br>Chemotherapy | Cervical lymph nodes | 4 × 6 | 500,000 | NR |
| 8 | Nasopharynx | $T_0N_3$ | Radiation<br>Chemotherapy | Cervical lymph nodes | 6 × 6 | 500 | NR |
| 9 | Larynx | $T_4N_0$ | Surgery<br>Radiation | Skin peritracheostomy | 5 × 4 | 500,000 | NR |
| 10 | Parotid gland | $T_4N_2$ | Radiation<br>Chemotherapy | Primary tumour<br>Cervical lymph nodes<br>Skin | 7 × 7[a] | 500,000 | NR |
| 11 | Hypopharynx | $T_3N_1$ | Radiation<br>Chemotherapy | Primary tumour | 2 × 2 | 500 | PR |
| 12 | Oropharynx | $T_2N_1$ | Radiation | Primary tumour | 2.5 × 4 | 500 | NR |
| 13 | Parotid gland | $T_2N_1$ | Surgery<br>Radiation<br>Chemotherapy | Cervical lymph nodes | 4 × 4 | 500,000 | NR |
| 14 | Larynx | $T_3N_1$ | Surgery<br>Radiation | Primary tumour<br>Cervical lymph nodes | 3 × 3<br>2.5 × 3 | 500,000 | MR |
| 15 | Oropharynx | $T_4N_1$ | Radiation<br>Chemotherapy | Cervical lymph nodes | 3.5 × 4 | 500 | MR |
| 16 | Larynx | $T_3N_2$ | Surgery<br>Radiation | Cervical lymph nodes | 4 × 5 | 500,000 | NR |
| 17 | Oral cavity | $T_2N_1$ | Surgery<br>Radiation<br>Chemotherapy | Primary tumour | 1.5 × 2.5 | 500 | PR |
| 18 | Hypopharynx | $T_3N_2$ | Radiation<br>Chemotherapy | Primary tumour | 3 × 3 | 500 | NR |
| 19 | Hypopharynx | $T_3N_1$ | Radiation<br>Chemotherapy | Primary tumour | 2 × 2.5 | 500 | MR |
| 20 | Oral cavity | $T_2N_1$ | Radiation<br>Chemotherapy | Primary tumour | 4 × 4 | 500,000 | NR |
| 21 | Hypopharynx | $T_3N_3$ | Radiation<br>Chemotherapy | Primary tumour<br>Cervical lymph nodes | 3 × 2<br>3 × 3 | 500 | MR |
| 22 | Larynx | $T_4N_1$ | Radiation<br>Chemotherapy | Primary tumour<br>Cervical lymph nodes | 5 × 6[a] | 500,000 | NR |
| 23 | Oral cavity | $T_4N_0$ | Surgery<br>Radiation<br>Chemotherapy | Primary tumour<br>Cervical lymph nodes | 3.5 × 3<br>3 × 4 | 500 | NR |
| 24 | Paranasal sinus | $T_3N_1$ | Radiation<br>Chemotherapy | Primary tumour<br>Cervical lymph nodes | 5 × 8[a] | 500,000 | NR |
| 25 | Larynx | $T_3N_0$ | Radiation<br>Chemotherapy | Primary tumour | 2.5 × 3 | 500,000 | NR |
| 26 | Oropharynx | $T_3N_1$ | Surgery<br>Radiation<br>Chemotherapy | Primary tumour<br>Cervical lymph nodes | 3 × 3<br>3.5 × 4 | 500,000 | MR |
| 27 | Oral cavity | $T_2N_1$ | Surgery<br>Radiation | Primary tumour<br>Cervical lymph nodes | 3.5 × 4<br>3 × 4 | 500,000 | MR |
| 28 | Hypopharynx | $T_4N_3$ | Radiation<br>Chemotherapy | Primary tumour<br>Cervical lymph nodes | 2.5 × 2.5<br>3 × 4 | 500 | MR |
| 29 | Oropharynx | $T_2N_1$ | Surgery<br>Radiation | Primary tumour | 3.5 × 3.5 | 500 | MR |
| 30 | Oropharynx | $T_3N_0$ | Radiation<br>Chemotherapy | Primary tumour | 4 × 4 | 500 | MR |
| 31 | Larynx | $T_3N_2$ | Surgery<br>Radiation<br>Chemotherapy | Cervical lymph nodes | 5 × 4 | 500,000 | NR |

[a]This measure includes primary tumour and lymph nodes.

steroidal anti-inflammatory drugs (except paracetamol) or any other drug during the experiment.

### *Patient evaluation*

General assessment prior to treatment included history and physical examination, haematological and blood chemistry examinations, urine examination, ECG and chest radiography. Other clinical examinations were undertaken to assess suspected sites of metastasis. Tumour status was determined by CT scan. Tumour size was expressed as the product of the two major perpendicular diameters, or as the sum of the products in the case of multiple lesions. Toxicity was evaluated according to the WHO criteria. Responses were

**Figure 1** Recurrence of a radiation-treated tumour of the left tonsillar fossa (patient 5). Left: The day before the beginning of IL-2 course. Right: Disappearance of the tumour 1 month after IL-2. T, tongue; PA, palatine arch; C, cheek. The arrowheads indicate the borders of the tumour mass.

**Figure 2** Recurrence of a tumour of the lateral floor of the mouth previously treated by surgery and radiotherapy (patient no. 17). Left: The day before the beginning of the IL-2 course. Right: Decrease and necrosis (white area) of the tumour 1 month after IL-2. T, tongue; C, cheek. The arrowheads indicate the border of the tumour mass.

rated as follows: CR, if the tumour was no longer evident; PR, if the sum of the products of the two major diameters of all lesions was reduced by more than 50%; minor response (MR), if the reduction was less than 50% but greater than 25%; no response (NR), absence of both response and progression; progression of the disease, appearance of new lesions or $>25\%$ increase in volume of one or more measurable lesions. Tumours were measured once a week by two operators independently, and the average value is reported. No significant differences in evaluation were found. Size was determined by CT or by physical examination with fibre-optic visualisation where necessary. A response was considered complete, partial or minor when the same measurements were found for two successive weeks and then lasting 1 month.

*Treatment plan*

Each patient was randomly assigned a dose of 500 or 500,000 IU of rIL-2 per day for administration diluted in 1 ml of physiological saline supplemented with 10% human serum albumin (HSA) (Institut Merieux, Lyon, France) to stabilise rIL-2, which is a markedly hydrophobic protein, and improve its absorption via the lymphatic system (Bocci *et al.*, 1985). Inoculation was performed in the morning with a 26 gauge needle at a depth of 15 mm in the anterior margin of the sternocleidomastoid muscle at 15 mm from its insertion on the mastoid (Cortesina *et al.*, 1988). The injections were performed on the same side as the recurrent tumour when draining lymph nodes were still present, and contralaterally when only contralateral lymph nodes were present, as in patients who had undergone unilateral neck dissection for oral cavity, oropharyngeal or parotid gland tumours. The patient was kept under observation for at least 30 min in the event of day-hospital administration. The ten daily injections were followed by an interval without treatment until day 40. A further three courses of ten injections were given at 30 day intervals in the absence of a CR. A Karnofsky index $>70\%$ was required prior to their commencement. Haematological and blood chemistry examinations were performed on day 0, day 10 and day 40. To ensure that injections were performed in the same way in both centres, the first patient in each location was treated by the same operator.

### Results

The results are reported in Table I. The CR was observed in a patient (no. 5) who had received radiotherapy for an oropharyngeal tumour. On recurrence after a 9-year disease-free interval, he refused chemotherapy and commenced the rIL-2 treatment. After 10 days, the tonsillar fossa began to be covered with a layer of necrosis. This then gradually disappeared to reveal smooth clean mucosa after 1 month (Figure 1). The four MR patients (nos. 15, 26, 29 and 30) were bearers of more extensive and more heavily treated recurrent oropharyngeal tumours who received IL-2 1 year or longer after the end of their previous therapy. MR was clearly evident after 30 days.

One PR and two MRs were obtained in the five patients with oral cavity tumours. The PR was observed in a patient (no. 17) with recurrence of a lateral tumour of the floor of the mouth, treated surgically and with radiotherapy 3 years earlier. The first recurrence appeared after an 18-month disease-free interval and was treated with chemotherapy for 6 months. The second recurrence appeared after a further year and was treated with IL-2. Necrosis of the tumour surface was noted after 12 days, and 60% reduction in size was reached after 60 days (Figure 2). The two MR patients (nos. 1 and 27) presented recurrences at the primary tumour site and in the cervical lymph nodes. Conventional management had been terminated 9 months prior to IL-2 therapy. A more than 50% reduction in size of the primary site recurrence was obtained in both cases after 20 days. The lymph node enlargement, however, was unaffected, and hence the results were classified as MR.

One PR and three MRs were found in five hypopharynx recurrences. The PR was observed in a patient (no. 11) who had received combined radio- and chemotherapy. The recurrence treated with IL-2 occurred after a 1 year disease-free interval. Direct laryngoscopy revealed its almost complete disappearance after 30 days. The MR patients (nos. 19, 21 and 28) had not been treated for 7 months. MRs were noted after 20 days.

One PR and three MRs were obtained in eight patients with laryngeal recurrences. The PR was observed in a patient (no. 2) who had undergone total laryngectomy for the primary tumour and radiotherapy for the first recurrence. The second recurrence (treated with IL-2) appeared after an 8 month disease-free interval. PR with improved deglutition was achieved after 15 days. The MRs were observed after 20 days in a patient (no. 3) 6 months after the conclusion of chemotherapy, and another (no. 14) 2 years after radiotherapy. The last MR was noted in one of the two subjects with recurrent parotid gland carcinoma. This patient (no. 6) had received various ineffective treatments. IL-2 was begun 6 months after the termination of chemotherapy. Flattening of the tumour and a $>40\%$ reduction in its size were observed after 25 days.

Clinical responses were nearly always preceded by improvement of the functional impairment caused by tumour infiltration of the upper respiratory-digestive organs. Pain appeared to increase to a slight degree at first, though it diminished or disappeared when tumour shrinkage began. No major toxic effects were noted in both IL-2 dose groups. Occasional fever episodes (more pronounced in patients receiving the higher dose) were easily controlled with paracetamol. In some cases, reduction of the tumour mass was accompanied by the almost complete disappearance of the necrotic component. Responses lasted 3–5 months and further IL-2 courses were ineffective (data not shown).

### Discussion

Perilymphatic administration of low-dose courses of rIL-2 is a simple manoeuvre, devoid of local and systemic side-effects. It led to one CR and three PRs in 31 patients (13%) with rH&NSCC. MRs are reported for interest only, since these carcinoma recurrences progress rapidly and fail to respond to standard therapy, and are omitted in the overall evaluation of the results. All these findings are in line with those of our previous studies conducted with nIL-2 (Cortesina *et al.*, 1988, 1991). Unfortunately, the brief duration of all responses and the ineffective treatment of further relapses observed in this and in our previous study point to the development of resistance to IL-2.

One interesting finding in this study is that doses of 500 U of rIL-2 infused regionally are effective, whereas 500,000 U doses are not. Interestingly, in a similar study using intra-arterial rIL-2 administration in rH&NSCC patients, the responses seen were at low dose of IL-2 in comparison with the high dose (Gore *et al.*, 1992), while in experimental models low doses are often more effective inducers of local reactivity (Forni *et al.*, 1985, 1987; Bosco *et al.*, 1990). However, the number of patients is too small for significant comparison. The efficacy of IL-2 treatment may be markedly influenced by the size of the recurrences and their histological features.

The best results in this and our previous study were obtained in small oropharyngeal recurrences. An encouraging susceptibility to IL-2 management was also displayed by hypopharyngeal tumours, as shown initially by marked regression of dysphagia and confirmed by endoscopy. The four responses were in small recurrences in the primary tumour site as opposed to the lymph nodes. A reason is thus found for the failure of nasopharyngeal carcinomas to respond to either nIL-2 or rIL-2, since their recurrences are usually located in the lymph nodes. In the case of lymph node and combined tumour site plus lymph node recurrence, MRs only were found.

The temporary activity of IL-2 against small rH&NSCC recurring at the site of the primary tumour would appear to be confirmed. In effect, the four responses were obtained in tumours that seemed unlikely to respond as they had already been pretreated and no longer responded to conventional therapy. The efficacy of the immune reactivity becomes marginal in these conditions. Patients with rH&NSCC display marked immunosuppression that may impair the establishment of systemic reactivity (Lundy *et al.*, 1974; Cortesina *et al.*, 1982; Morra *et al.*, 1984; Wolf *et al.*, 1986). The fact that a biological therapy was also able to induce 12 MRs is a further point of interest. Our previous findings with nIL-2 (Cortesina *et al.*, 1988, 1991) and present findings with rIL-2 display some common features: good susceptibility on the part of small recurrences in the primary tumour site, especially the oropharynx; the absence of side-effects; the functional effectiveness of the administration route; and the inverse proportion between tumour mass and clinical response. These features suggest that small primary head and neck tumours before surgery and the minimal residual disease that follows surgery are more suitable settings for locoregional immunotherapy. The aim is to include the injection of IL-2 around tumour-draining lymph nodes in multidisciplinary strategies designed to improve the prognosis of patients with H&NSCC low 5-year survival percentages.

This work was supported by the Italy-USA Program on Cancer Therapy, AIRC and CNR PF 'ACRO'. The authors wish to thank Dr Iliffe for his careful review of the manuscript.

### References

BOCCI, V. (1985). Reduction and role of interferon in physiological condition. *J. Biol. Response Modif.*, **4**, 340–344.

BOSCO, M.C., GIOVARELLI, M., FORNI, M., MODESTI, A., SCARPA, S., MASUELLI, L. & FORNI, G. (1990). Low doses of IL-4 injected perilymphatically in tumour bearing mice inhibit the growth of poorly and apparently non immunogenic tumours and induce a tumor-specific immune memory. *J. Immunol.*, **145**, 3136–3143.

COLOMBO, M.P., MODESTI, A., PARMIANI, G. & FORNI, G. (1992). Local cytokine availability elicits tumor rejection and systemic immunity through granulocyte–T lymphocyte cross-talk. *Cancer Res.*, **52**, 4853–4857.

CORTESINA, G., CAVALLO, G.P., BEATRICE, F., SARTORIS, A., BUSSI, M., MORRA, B., DI FORTUNANTO, V., POGGIO, E. & RENDINE, S. (1982). Production of leukocyte migration inhibition factor by lymphocytes of larynx cancer patients stimulated by laryngeal carcinoma solubilized membrane antigens. *Tumori*, **68**, 39–44.

CORTESINA, G., DE STEFANI, A., GIOVARELLI, M., BARIOGLIO, M.G., CAVALLO, G.P., JEMMA, C. & FORNI, G. (1988). Treatment of recurrent squamous cell carcinoma of head and neck with low doses of interleukin-2 injected perilymphatically. *Cancer*, **62**, 2482–2485.

CORTESINA, G., DE STEFANI, A., GALEAZZI, E., CAVALLO, G.P., JEMMA, C., GIOVARELLI, M., VAI, S. & FORNI, G. (1991). Interleukin-2 injected around tumor draining lymph nodes in head and neck cancer. *Head Neck*, **13**, 125–131.

FORNI, G., GIOVARELLI, M. & SANTONI, A. (1985). Lymphokine activated tumor inhibition *in vivo*. The local administration of interleukin-2 triggers non reactive lymphocytes from tumor bearing mice to inhibit tumor growth. *J. Immunol.*, **134**, 1305–1311.

FORNI, G., GIOVARELLI, M., SANTONI, A., MODESTI, A. & FORNI, M. (1987). Interleukin-2 activated tumor inhibition *in vivo* depends on the systemic involvement of host immune reactivity. *J. Immunol.*, **138**, 4033–401.

GORE, M.E., RICHES, P., MACLENNAN, K., O'BRIEN, M., MOORE, J., DADIAN, G., LORENTZOS, A., GARTH, R., MOSCOVIC, E., ARCHER, D., BREACH, N., HENK, M., RHYS-EVANS, P. & KING, D.M. (1992). Phase I study of intra-arterial interleukin-2 in squamous cell carcinoma of the head and neck. *Br. J. Cancer*, **66**, 405–407.

LUNDY, J., WANEBO, K., PINSKY, C., STRONG, E. & OETTGEN, H. (1974). Delayed hypersensitivity reactions in patients with squamous cell cancer of the head and neck. *Am. J. Surg.*, **128**, 530–533.

MORRA, B., BEATRICE, F., CAVALLO, G.P., BUSSI, M., DI FORTUNATO, V., POGGIO, E., VERCELLINO, M., SARTORIS, A. & CORTESINA, G. (1984). Evaluation of blocking mechanisms against immunological response in patients with laryngeal carcinoma. *Laryngoscope*, **94**, 825–828.

PERICLE, F., DI PIERRO, F. & FORNI, G. (1992). Clinical trials with local administration of haematopoietic growth factors II. In *Lymphohaematopoietic Growth Factors in Cancer Therapy II*. Mertelsmann, R. (ed.), pp. 234–244. Springer: Berlin.

RECOMMENDATIONS FROM THE DECLARATION OF HELSINKI (1985). *Fed. Proc.*, **47**, 17.

RIVOLTINI, L., GAMBACORTI-PASSERINI, C., SQUADRELLI-SARACENO, M., GROSS, M., CANTU', G., MOLINARI, R., ORAZI, A. & PARMINANI, G. (1990). *In vivo* interleukin 2-induced activation of lymphokine-activated killer cells and tumor cytotoxic T-cells in cervical lymph nodes of patients with head and neck tumors. *Cancer Res.*, **50**, 5551–5557.

WOLF, G.T., HUDSON, J.I., PETERSON, K.A., MILLER, H.L. & MCCLATCHEY, K.D. (1986). Lymphocyte subpopulations infiltrating squamous carcinoma of the head and neck: correlation with extent of tumor and prognosis. *Otolaryngol. Head Neck Surg.*, **95**, 142–152.